# Reduced maternal immunity and vertical transfer of immunity against SARS-CoV-2 variants of concern with COVID-19 exposure or initial vaccination in pregnancy

**DOI:** 10.3389/fimmu.2023.1216410

**Published:** 2023-09-11

**Authors:** Rupsa C. Boelig, Sidhartha Chaudhury, Gregory D. Gromowski, Sandra Mayer, Jocelyn King, Zubair H. Aghai, Elke Bergmann-Leitner

**Affiliations:** ^1^ Department of Obstetrics and Gynecology, Division of Maternal Fetal Medicine, Sidney Kimmel Medical College, Thomas Jefferson University, Philadelphia, PA, United States; ^2^ Department of Pharmacology, Physiology, and Cancer Biology, Sidney Kimmel Medical College, Thomas Jefferson University, Philadelphia, PA, United States; ^3^ Center for Enabling Capabilities, Walter Reed Army Institute of Research, Silver Spring, MD, United States; ^4^ Viral Diseases Branch, Walter Reed Army Institute of Research, Silver Spring, MD, United States; ^5^ Division of Neonatology, Department of Pediatrics, Nemours, Philadelphia, PA, United States; ^6^ Immunology Core, Biologics Research & Development, Walter Reed Army Institute of Research, Silver Spring, MD, United States

**Keywords:** COVID-19, COVID vaccine, SARS-CoV-2, pregnancy, variants of concern, maternal immunity, neonatal immunity

## Abstract

**Introduction:**

As the SARS-CoV-2 pandemic continues to evolve, we face new variants of concern with a concurrent decline in vaccine booster uptake. We aimed to evaluate the difference in immunity gained from the original SARS-CoV-2 mRNA vaccine series in pregnancy versus SARS-CoV-2 exposure during pregnancy against recent variants of concern.

**Study Design:**

This is a retrospective analysis of previously collected samples from 192 patients who delivered between February 2021 and August 2021. Participants were categorized as 1) COVID vaccine: mRNA vaccine in pregnancy, 2) COVID-exposed, and 3) controls. The primary outcome was neutralizing capacity against wild-type, Delta, and Omicron-B1 between cohorts. Secondary outcomes include a comparison of cord-blood ID50 as well as the efficiency of vertical transfer, measured by cord-blood:maternal blood ID50 for each variant.

**Results:**

Pregnant women with COVID-19 vaccination had a greater spike in IgG titers compared to both those with COVID-19 disease exposure and controls. Both COVID exposure and vaccination resulted in immunity against Delta, but only COVID vaccination resulted in significantly greater Omicron ID-50 versus controls. The neutralizing capacity of serum from newborns was lower than that of their mothers, with COVID-vaccination demonstrating higher cord-blood ID50 vs wildtype and Delta variants compared to control or COVID-exposed, but neither COVID-exposure nor vaccination demonstrated significantly higher Omicron ID50 in cord-blood compared to controls. There was a 0.20 (0.07-0.33, p=0.004) and 0.12 (0.0-0.24, p=0.05) increase in cord-blood:maternal blood ID50 with COVID vaccination compared to COVID-19 exposure for wild-type and Delta respectively. In pair-wise comparison, vertical transfer of neutralization capacity (cord-blood:maternal blood ID50) was greatest for wild-type and progressively reduced for Delta and Omicron ID50.

**Conclusion:**

Pregnant patients with either an initial mRNA vaccination series or COVID-exposure demonstrated reduced immunity against newer variants compared to wild-type as has been reported for non-pregnant individuals; however, the COVID-vaccination series afforded greater cross-variant immunity to pregnant women, specifically against Omicron, than COVID-disease. Vertical transfer of immunity is greater in those with COVID vaccination vs COVID disease exposure but is reduced with progressive variants. Our results reinforce the importance of bivalent booster vaccination in pregnancy for both maternal and infant protection and also provide a rationale for receiving updated vaccines as they become available.

## Introduction

The COVID-19 pandemic continues to impact global health, including in the United States, and has a disproportionate effect on pregnant women and children. Pregnant women have been at higher risk for severe morbidity and mortality from COVID-19 (1–3) (CDC); pregnancies with COVID face an increased rate of pregnancy complications, including preeclampsia, preterm birth, and stillbirth (4). In addition to the neonatal impact of maternal COVID-19 as a result of prematurity (5), COVID-19 infection has a significant effect on pediatric health. Exposure risk in infants is due to contact with other family members, which is primarily their mother. Early infant immunity is dependent on passively acquired immunity through placental transfer/breastmilk following maternal vaccination or exposure. Even in the setting of infection of more recent variants of concern (Omicron), there remains an increased rate of adverse maternal and neonatal outcomes in the setting of maternal COVID disease, although the severity was progressively attenuated with vaccination and booster vaccination (6). Despite these documented benefits of vaccination, and booster dosing, as of July 2023, approximately 75% of pregnant individuals nationally had completed the primary COVID vaccine series, but only 14% had received the updated bivalent booster dose (7). This is consistent with general trends in the non-pregnant adult population. A recent study found that 15-17% of individuals < 45 years old have received the bivalent booster, and ~20% do not plan to get the bivalent booster at all (8). Given the poor uptake of the bivalent booster, we aimed to compare the neutralization capacity of immune serum against wild-type, Delta, and Omicron variants in those who had received the original vaccine series or had a history of COVID-disease. Secondarily, we also aimed to identify how maternal immunity and time from exposure were associated with placental transfer and infant (cord-blood) immunity. Performing these cross-reactivity assessments on samples collected during a phase of the pandemic when immunity could be clearly attributed to COVID-exposure involving few viral lineages versus the monovalent vaccines can reveal qualitative differences in the resulting immunity.

## Materials and methods

This is a secondary analysis of samples collected for a previously published study on the impact of COVID-19 disease and vaccination on the maternal-fetal unit (9). Of the 306 participants in that study, there were 192 samples available for additional analyses. As previously described, these were patients enrolled in an ongoing delivery biorepository, and the study involved the collection of maternal and cord-blood at the time of delivery (9, 10). The objective was to compare maternal and neonatal (cord-) blood for their neutralizing capacity against Omicron and other SARS-CoV-2 variants in study participants who had received the original COVID-19 mRNA vaccine or had a recent history of COVID-19 disease. All those in the vaccine category had received both doses of either the Pfizer or Moderna vaccines, although the exact vaccine type was not recorded. None of these participants had received the newly available bivalent booster yet, thus allowing us to definitively determine cross-reactivity with variants of concern.

### Cohort classification

Participants were binned into the following groups: 1) COVID-19 vaccination without history of COVID-19 disease, 2) COVID-exposure: history of COVID-19 in the pregnancy or seropositive based on SARS-CoV-2 nucleocapsid IgG titers (10), and 3) control: no history of COVID vaccination, or COVID disease (confirmed both by history and seronegative for SARS-CoV-2 nucleocapsid).

### Data collection

Electronic medical records were reviewed for demographic, medical, and obstetric history, date of first positive SARS-CoV-2 PCR test, date of delivery, antenatal complications, and delivery outcomes. Standard of care at our institution includes a universal SARS-CoV-2 PCR test on admission and a neonatal SARS-CoV-2 PCR test if the mother had a positive diagnosis on admission for delivery.

### Serology

For assessing antibody specificity, a multiplex testing platform (MesoScale Diagnostics, Rockville, MD) was employed: antigens were manufactured in a mammalian expression system (Expi 293 F) are printed onto 10-plex plates. The antigens used were: HA-trimer from Influenza A (Hong Kong H3), spike (soluble ectodomain with T4 trimerization domain) trimers from SARS-CoV-2, SARS-CoV-1, MERS-CoV, and the betacoronaviruses HKU-1 and OC43, as well as the spike N-terminal domain (NTD, Q14-L303 of the SARS-CoV-2 spike sequence), receptor binding domain (RBD, R319-F541 of the SARS-CoV-2 spike sequence), and nucleocapsid protein (N; full length) for SARS-CoV-2, and bovine serum albumin (BSA, as negative control). Assays were performed following the manufacturer’s instructions. In brief, plates were blocked using Blocker A Solution and incubated at room temperature (RT) for 1h on a plate shaker at 700 rpm. The plates were washed three times with 1x MSD Wash Buffer. Sera were diluted to 1:1000 with Diluent 100. Positive samples (pooled human serum from COVID-19 patients) and negative samples (pooled pre-pandemic human serum) were used as controls. Plates were sealed and incubated at RT for 2h on a plate shaker at 700 rpm, then washed three times with 1x MSD Wash Buffer. The detection antibody, SULFO-TAG anti-human IgG or anti-human IgM antibody was diluted to 2 µg/ml in Diluent 100 (MSD), added to the wells, and incubated at RT for 1h on a plate shaker 800rpm. After washing, MSD GOLD Read Buffer B was added to each well and the plates were read immediately on the MESO QuickPlex SQ 120 (MSD).

### Neutralization capacity

This assay has been described previously (11). The Spike expression plasmid sequences for SARS-CoV-2 were codon optimized and modified to remove the last 18 amino acids of the cytoplasmic tail, which improves S incorporation into pseudovirions (PSV). PSV were produced by cotransfection of HEK293T/17 cells with either a SARS-CoV-2 S plasmid based on the Wuhan-Hu-1 genome sequence (GenBank accession number MN908947.3) and an HIV-1 pNL4-3 luciferase reporter plasmid (pNL4-3.Luc.R-E-, NIH HIV Reagent Program, catalog number 3418). S expression plasmids for SARS-CoV-2 VOCs were similarly codon optimized and modified and included the following mutations: B.1.617.2/Delta (E156G, D614G, P681R, T19R, T478K, L452R, D950N, 157-158 del), B.1.1.529/Omicron (A67V, 69-70 del, T95I, G142D, 143-145 del, N211I, 212 del, G339D, S371L, S373P, S375F, S477N, T478K, E484A, Q493R, G496S, Q498R, N501Y, Y505H, T547K, D614G, H655Y, N679K, P681H, N764K, D796Y, N856K, N969K, Q954H, L981F). Infectivity and neutralizing titers were determined using ACE2-expressing HEK293 target cells (Integral Molecular). Test sera were serially diluted, mixed with an equal volume of diluted PSV, and plates were incubated for 1 h at 37°C. Target cells were added to each well (40,000 cells per well), and plates were incubated for an additional 48 h. Relative light units were measured with the Synergy Neo2 Hybrid Multi-Mode Microplate Reader (Agilent BioTek) using the Bright-Glo Luciferase Assay System (Promega). Neutralizationdose–response curves were fitted by nonlinear regression using GraphPad Prism, and titers are reported as the reciprocal of the serum dilution necessary to achieve 50% inhibition of SARS-CoV-2 infectivity (ID50).

### Outcomes

The primary outcome was neutralizing capacity, measured by the reciprocal of the serum dilution necessary to achieve 50% inhibition of SARS-CoV-2 infectivity (ID50) against wild-type, Delta, and Omicron-B1 between cohorts. Secondary outcomes include a comparison of cord-blood ID50 as well as efficiency of vertical transfer, measured by cord-blood:maternal blood ID50 for each variant, correlation between titers and variant-specific immunity, and factors associated with maternal ID50.

### Statistical plan

Statistical analysis was conducted using SPSS v. 26.0 (Chicago, SPSS, Inc) and R. Regarding baseline characteristics- categorical comparisons were done with chi-square analysis or Fisher’s exact test as appropriate and continuous variables were compared with one-way ANOVA. Serological titers and ID-50 values were log-transformed, and compared with ANCOVA, unadjusted, and adjusted taking into consideration potential covariates that were identified to be different between groups at p<0.05. Correlation between latency, titers, and ID50 was assessed with bivariate correlation and reported with Pearson correlation coefficient (r). Comparisons of paired maternal and cord-blood serology were carried out using linear regression to determine the slope, correlation coefficient, and R (2). P<0.05 is considered significant for all analyses. Pair-wise comparison of the difference in variant-specific immunity was done with the Wilcox Rank Sum test. Figures were generated using the *SPSS*, and *stats*, *ggplot2*, and *corrplot* packages in R.

## Results

### Cohort description

There were 192 participants who had samples available for additional analyses. They delivered between February 2021-August 2021. This time period includes the Delta wave, which began in India in late 2020, emerged in the United States in April 2021, and dominated in the summer of 2021. This time period does not include the Omicron variant wave, which was first identified in South Africa in November 2021, and quickly spread around the world. Analyzing this sample set provides insights into how future variants from lineages divergent from the vaccine-included sequences will perform and make the results of this study relevant. There were 88 in the control group, 57 in the COVID-exposed group, and 47 in the COVID-vaccine group. Of 57 in the COVID-exposed group, there were 28 participants who did not have a documented COVID-19 infection but were seropositive. Maternal-cord-blood dyads at delivery were available for 114 participants, N=54 control, N=32 COVID-exposed, and N=28 COVID- vaccine. There were some significant baseline differences between groups, including race, maternal age and maternal BMI, and time from exposure (**Table 1**). All of those in the vaccine group had received both doses of an mRNA-based COVID vaccine and were >7 days from the first vaccine dose. Of those in the COVID-exposed group, only 19 had a specific date recorded for their COVID-19 infection and the majority of these were >7 days from delivery (**Table 1**).

**Table 1:** Characteristics of cohort.

Characteristics	COVID-Vaccine N=47	COVID-Exposed N=57	Control N=88	p-value
Race				
White	33 (70.2%)	8 (14.0%)	34 (38.6%)	<0.001
Black	4 (8.5%)	30 (52.6%)	36 (40.9%)	
Asian	6 (12.8%)	3 (5.3%)	8 (9.1%)	
Hispanic	0 (0%)	13 (22.8%)	7 (8.0%)	
Other	4 (8.5%)	3 (5.3%)	3 (3.4%)	
Prior full-term delivery	23 (48.9%)	31 (54.4%)	53 (60.2%)	0.44
Prior preterm birth	24 (12.5%)	9 (15.8%)	12 (13.6%)	0.32
Twin gestation	2 (4.3%)	1 (1.8%)	2 (2.3%)	0.33
Maternal age	34.3 ± 4.4	29.0 ± 5.9	31.2 ± 5.5	<0.001
Maternal BMI	30.5 ± 6.3	35.9 ± 7.2	34.5 ± 6.9	<0.001
Gestational age at delivery (weeks)	38.4 ± 1.5	38.6 ± 1.2	38.5 ± 1.3	0.74
Time from vaccine or COVID disease (days)	85.5 ± 44.3 (N=47)	91.6 ± 73.8 (N=19)	Not applicable	0.68
Delivery > 7 days from vaccine or COVID disease	47 (100%) (N=47)	16 (84.2%) (N=19)		0.006

### SARS CoV-2 serological titers

**Figure 1:**
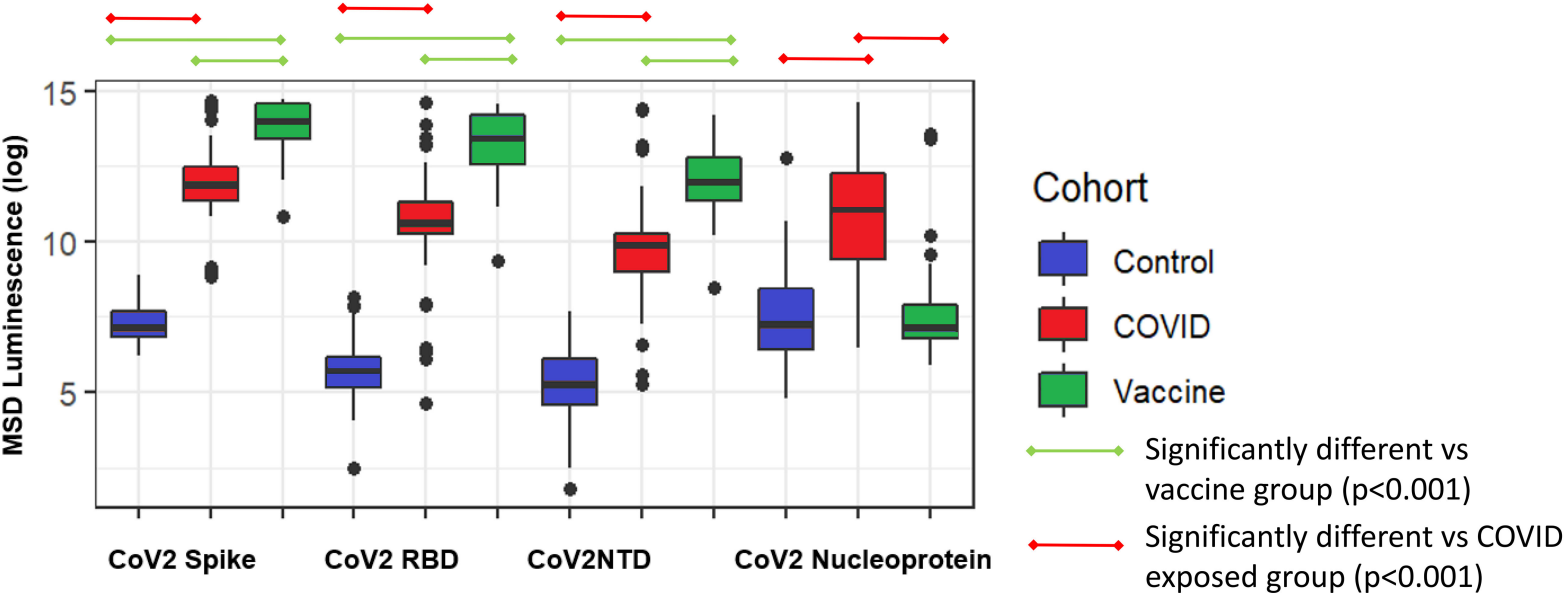
Comparison of SARS-CoV-2 antibody epitopes in control (N=88), COVID exposed (N=57), and COVID-vaccinated subjects(N=47). Spike, full-length spike protein; RBD, spike receptor binding domain; NTD, N-terminal domain; Nucleoprotein, nucleocapsid.

COVID-19 vaccination was associated with higher mean full-length spike IgG titers compared to those with COVID-19 disease and controls. Those with COVID-19 exposure had lower titers compared to the vaccine group. Participants with COVID exposure had, as expected, higher nucleocapsid titers than controls and COVID vaccine recipients (**Figure 1**).

### Neutralizing capacity (ID50) against variants of concern

**Figure 2:**
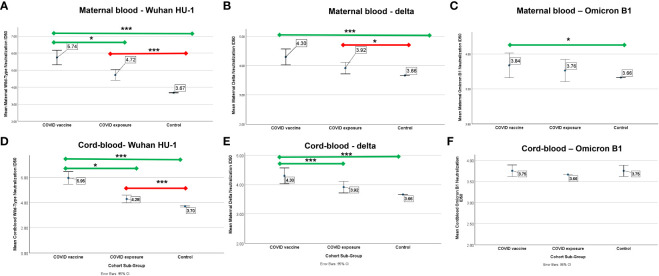
Mean neutralization ID50 (log) against wild-type, Delta, and Omicron B-1 variants in pregnant patients who had COVID-19 (N=57), COVID-vaccine (N=47), or neither (control, N=88). Error bars indicate a 95% confidence interval. Comparison of mean ID50 (log) by cohort subgroup against **(A)** wild-type, **(B)** Delta, and **(C)** Omicron B1 variants in maternal blood and **(D)** wild-type, **(E)** Delta, and **(F)** Omicron B1 variants in cord-blood. Comparisons adjusted for age, race, BMI, and, between COVID-exposed and vaccine cohorts, time from infection. The green line indicates a comparison to COVID-vaccine, the red line indicates a comparison to COVID-disease. * p<0.05 *** p<0.001.

Those with COVID-19 mRNA vaccination had higher ID50 against wildtype and Delta virus, and to a lesser degree, Omicron, compared to controls in unadjusted and adjusted analyses. In contrast, those with COVID-exposure did not have any improved immunity against Omicron compared to controls (**Figure 2**). In pairwise comparisons for those who had received the COVID vaccine, there was progressively reduced ID-50 from wild-type (5.74 ± 1.45) to delta (4.30 ± 0.92) to Omicron (3.83 ± 0.60) (p<0.001 for all comparisons). For those with COVID exposure, there was progressively reduced ID-50 with Delta (3.91 ± 0.75) and Omicron (3.76 ± 0.60) compared to wild-type (4.7 ± 1.19) (p<0.001, p<001), and with Omicron compared to Delta (p=0.02).

**Figure 3:**
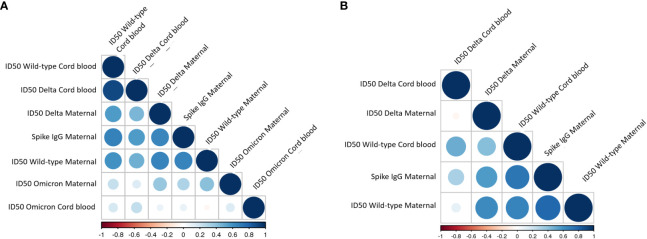
Correlation between antibody titer and neutralization capacity in maternal and cord-blood in **(A)** COVID vaccine cohort and **(B)** COVID exposed cohort. The color and size of the circles within the correlation matrix are corresponding to pairwise Pearson correlation coefficients. The factors in the individual matrices are ordered according to the degree of association between variables. Due to a lack of correlation with Omicron ID50, this was removed from the correlation matrix in B.

Among those with COVID-19 exposure or vaccination, time from exposure (days) was negatively correlated with neutralizing capacity for both wild-type (r=-0.35, p=0.005) and Delta (-0.31, p=0.01). Maternal full-length spike IgG titers were positively correlated with neutralizing capacity against wild-type (r=0.74, p<0.001), Delta (r=0.56, p<0.001) and Omicron-B1 (r=0.30, p=0.003) variants (**Figure 3**). The correlation matrices demonstrated the distinct relationships between antibody titers and neutralizing activities in the COVID vaccine vs. COVID disease cohorts: the COVID vaccine group showed a strong correlation between maternal spike titers and wild-type and Delta ID50 in maternal and cord-blood. Moreover, a weak correlation between maternal Omicron ID50 and maternal spike titers and wild-type and Delta neutralization capacity was observed (**Figure 3A**). In contrast, strong correlations in the COVID exposure cohort were only seen between maternal spike titers vs. wild-type and Delta neutralizing capacity. This correlation for antibody titer and neutralizing capacity in cord blood was strong for wildtype, but already diminished for Delta. Due to a lack of correlation with Omicron ID50, this analysis was removed from the correlation matrix in **Figure 3B**. In summary, the COVID vaccine cohort reveals more cross-reactivity against variant strains, including even some activity against Omicron. The latter was not observed in the COVID exposure cohort.

In a multivariable logistic regression analysis that takes into consideration race, age, BMI, latency, along with cohort sub-group, COVID-vaccination was positively associated with wild-type (B=1.3 (0.4 – 2.1), p=0.005), Delta (B=0.5 (0.1-1.1), p=0.047), and Omicron (B=0.4 (0.1-0.7), p=0.01) ID50 compared to COVID-exposed. These results are limited to those with time from exposure data available (**Table 1**).

### Vertical transfer of immunity

**Figure 4:**
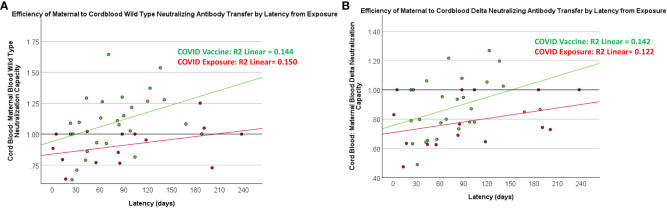
Relationship between latency and maternal-blood:cord-blood transfer of neutralization capacity against wild-type and Delta variants. COVID exposure data points and regression line represented in red. COVID vaccine data points and regression line represented in green.

**Figure 5:**
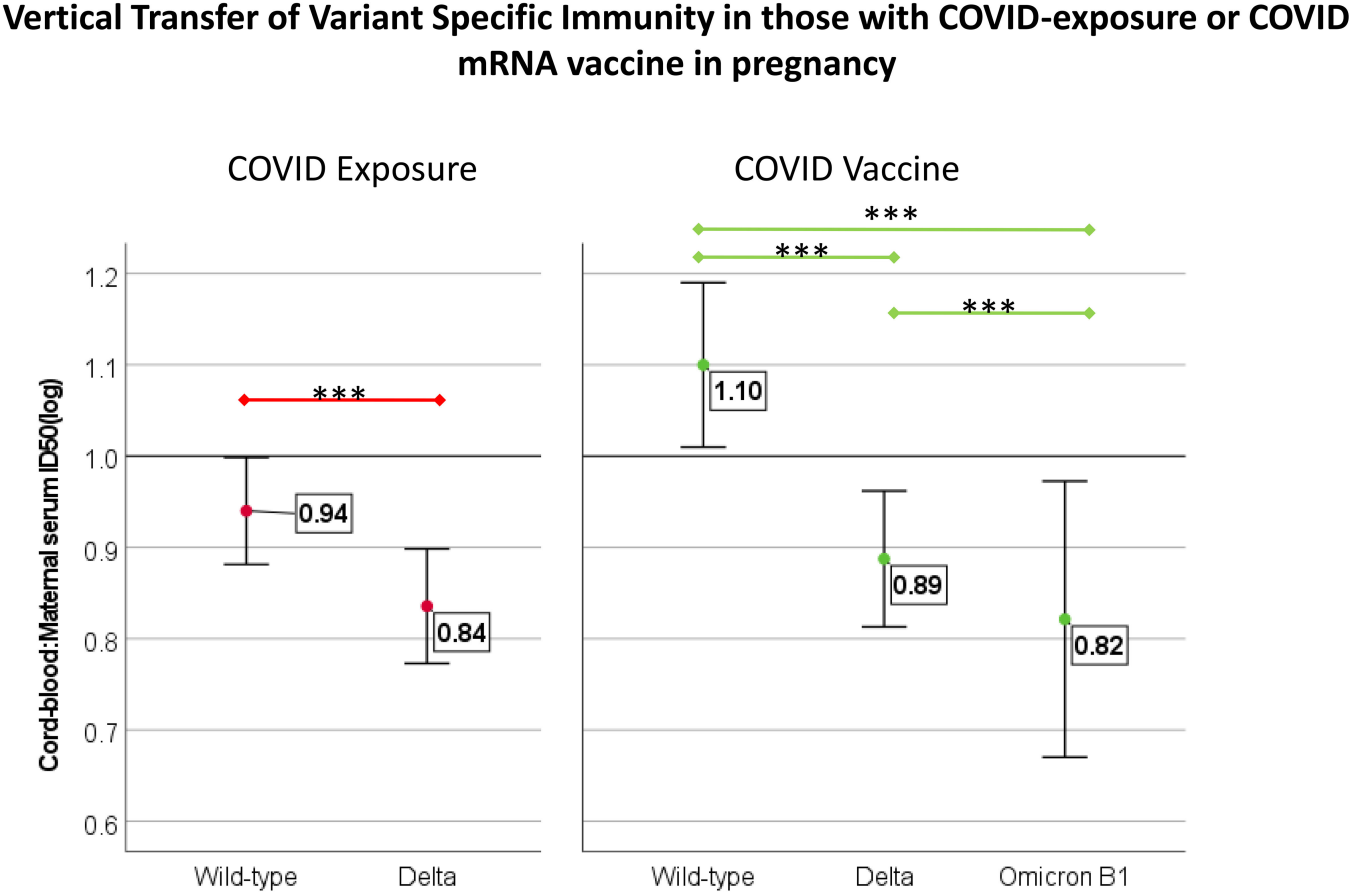
Comparison of transfer efficiency of immunity against variants of concern in those with COVID-vaccine (N=47) and COVID exposure (N=28) with maternal/cord-blood pair available. Transfer efficiency was reported as the ratio of cord-blood:maternal-blood ID50 for each variant. Comparison completed with Wilcox rank-sum test, *** indicates p<0.001. For those with COVID-disease, Omicron is not included due to a lack of increased neutralization capacity compared to controls.

There was a positive association between maternal and cord-blood titers for both COVID vaccine and COVID-19-exposed cohorts (**Figure 3**). Although overall neutralizing capacity decreased with increased time from exposure, the efficiency of transfer of neutralizing capacity increased with time from exposure (**Figure 4**). In a linear regression model, both time from exposure and COVID vaccination vs disease were positively associated with increased vertical transfer efficiency for the wild-type and the Delta variant. There was a 0.20 (0.07-0.33, p=0.004) and 0.12 (0.0-0.24, p=0.05) increase in cord-blood:maternal neutralizing capacity with COVID vaccination compared to COVID-19 exposure for wild-type and Delta neutralizing capacity respectively. Finally, in comparing the efficiency of vertical transfer of immunity to variants, the Wilcox rank sum comparison found that there was reduced efficiency from wild-type to Delta to Omicron (p<0.001); and among the COVID-exposure cohort reduced efficiency from wild-type to Delta variants (Omicron not evaluated due to lack of significant immunity against Omicron in the COVID-exposed cohort) (**Figure 5**).

## Discussion

Our results add the following three key points to the current body of knowledge regarding COVID vaccination in pregnancy: 1) Immunity induced by the original mRNA-based COVID vaccine is reduced against newer variants compared to the wild-type virus; this trend was even more marked when immunity was induced by infection rather than vaccination; 2) an initial COVID-vaccination series afforded greater cross-variant immunity, specifically against Omicron, than COVID-disease; 3) vertical transfer of immunity to newborns is not equivalent across variants. This suggests that the types of antibodies that provide cross-variant maternal immunity are limited in their transplacental passage. For example, IgM shows very limited placental transfer, but can vary by inflammatory status (12). While IgG antibodies as a whole have a high placental transfer efficiency, there is variable transfer efficiency within IgG isotypes, and this may also explain the variation in variant-specific immunity (13). Our results highlight the importance of maintaining immunity through vaccinations against SARS-CoV-2, the potential need for updating the vaccine as new variants develop, and the limitations of neonatal-acquired immunity.

### Results in the context of what is known

The maternal and neonatal benefits of COVID vaccination are well documented in the form of both reduced maternal morbidity and mortality as well as reduced infant COVID-related morbidity and mortality in the setting of initial vaccination series and earlier variants (14, 15). A recent study on patients boosted in the third trimester receiving the bivalent booster demonstrated increased Omicron spike titers compared to primary vaccination (16) but did not compare Omicron neutralizing capacity with prior COVID exposure. An earlier study evaluated the efficacy of COVID vaccination against wild-type and Alpha and Beta variants and found reduced responses against the Alpha and Beta variants, but did not include a comparison to control or disease exposure (17), and provided no data for the Delta or Omicron variants.

Our work adds to the current body of knowledge by evaluating how well an initial vaccination series, or early COVID exposure, protects against current variants of concern. Due to the size and heterogeneity of our cohort, we were able to study individuals with a range of time from infection and adjust for differences in baseline characteristics. Consistent with earlier studies comparing the COVID vaccine and disease exposure and wild-type immunity (18), we found vaccination afforded improved immunity, although attenuated, against new variants as well. Interestingly, we found reduced neonatal immunity against progressive variants, reflecting the limitations of passively acquired cross-variant immunity. This highlights the need to promote the use of variant-specific booster immunizations in pregnancy if not just for maternal benefit, but also for neonatal protection.

### Implications for research

Our results have several important implications for research. First, given the continued, global circulation of SARS-CoV-2 exposure, comparing vaccine-induced immunity to disease-induced immunity and the impact of vaccination and/or exposure on the immunological profile hold tremendous value. Second, our results highlight the limited utility of serological titers alone when analyzing immunity against newer variants of concern. Future research on SARS CoV-2 immunity should focus on neutralizing the capacity of immune serum and not just serological titers. For example, COVID-19 infections resulted in broad antibody response to SARS-CoV-2 spike protein and nucleocapsid, but, unlike vaccination, resulted in negligible Omicron neutralizing activity in both maternal and cord-blood sera. Third, we noted significantly higher neutralizing capacity against wild-type in cord-blood compared to maternal blood. The efficiency of vertical transfer of immunity is an important area for future research to optimize maternal immunization with the potential for neonatal benefit. This transfer, however, did not translate into equivalent protection against the Omicron variant, sub highlighting the distinct nature of the passive immunity acquired by neonates and the need for further research into whether, for example, the lack of IgM, or preference of transfer of specific IgG isotypes results in the difference in neutralizing capacity between maternal and cord-blood. This has important implications for the degree of functional neonatal immunity afforded by maternal vaccination, and optimal time periods of maternal vaccination for infant immunity. Fourth, the observed differences in the persistence of neutralizing antibody titers induced by vaccination vs SARS-CoV-2 exposure in maternal sera and the corresponding neutralizing activity in cord-blood strongly support the recommendation to vaccinate pregnant patients. Finally, the observation that 28% of control subjects were seropositive already by mid-2021 confirms the increasing endemicity of SARS-CoV-2 infections and the importance of taking existing immunity into consideration in future studies.

### Implications for clinical care

Our results have a number of important implications for clinical care. First and foremost, we highlight the importance of bivalent boosters during pregnancy; an initial vaccine series resulted in reduced maternal and no neonatal functional immunity against the recent variant of concern, i.e., Omicron. Poor bivalent booster uptake may be related to a false sense of security from the initial vaccine course or prior infection, and this data provides evidence to counter those assumptions. Second, our findings regarding the benefit of vaccination and latency in the vertical transfer of immunity, and the limited acquired cross-variant immunity, have important implications for the timing of maternal booster doses in pregnancy for the purpose of inducing infant immunity.

## Conclusion

In pregnant patients, an initial COVID mRNA vaccination series provides greater cross-variant protection and improved vertical transfer of immunity compared to COVID-exposure in a similar time period, however even with vaccination, there is reduced maternal immunity and vertical transfer of immunity against the recent variant of concern, Omicron. This highlights the importance of updated booster vaccination in pregnancy for both maternal immunity and acquired neonatal immunity and the need for further research into the specific antibodies that contribute to cross-variant immunity. The identification of conserved epitopes that lead to cross-strain reactivity and protection will be crucial for future vaccine design.

## Data availability statement

The original contributions presented in the study are included in the article/supplementary materials. Further inquiries can be directed to the corresponding author.

## Ethics statement

The studies involving humans were approved by Thomas Jefferson University Hospital. The studies were conducted in accordance with the local legislation and institutional requirements. Written informed consent for participation in this study was provided by the participants.

## Author contributions

RB and EB-L developed the study design, oversaw the analysis, and drafted the manuscript. EB-L conducted the serological titer assays and analyses. SC contributed to study design, statistical analyses, and manuscript drafting. GG, SM, and JK contributed to the study design, conducted the neutralization assays, and contributed to manuscript drafting. ZHA contributed to study design, interpretation of results, and manuscript drafting. All authors contributed to the manuscript, approved the final submitted version for publication, and are accountable for the content.

## Author disclaimer

Material has been reviewed by the Walter Reed Army Institute of Research. There is no objection to its presentation and/or publication. EB-L is a government employee and this work was prepared as part of her official duties. Title 17 U.S.C. § 105 provides that copyright protection under this title is not available for any work of the U.S. Government. The opinions or assertions contained herein are the private views of the authors and are not to be construed as official, or as reflecting the views of the Department of the Army or the Department of Defense. The investigators have adhered to the policies for the protection of human subjects as prescribed in AR 70-25. This paper has been approved for public release with unlimited distribution.
